# Second Pan-American Medical Congress

**Published:** 1896-06

**Authors:** 


### Second Pan-American Medical Congress.

The Committee on Organization of the Second Pan-American Medical Congress at Mexico City, Mexico, have notified, through their Secretary, Dr. Eduardo Liceaga, Dr. H. L. E. Johnson that he had been appointed the Vice-President of the Congress for the District of Columbia. Local organization will commence at once.

